# The sources of information of the genealogical tourist: the influence of social networks and genealogical associations

**DOI:** 10.1016/j.heliyon.2022.e11551

**Published:** 2022-11-10

**Authors:** Ricardo Urrestarazu-Capellán, José Correa-Fernández, Francisco Sánchez-Cubo

**Affiliations:** a Facultad de Ciencias Económicas y Empresariales, University of Málaga, C/El Ejido, 6, 29071, Málaga, Spain; b Department of Political Economy and Public Finance, Economic and Business Statistics and Economic Policy, University of Castilla-La Mancha, Cuenca, Spain

**Keywords:** Genealogical tourism, Tourist promotion, Sources of tourist information, Social networks, And associations

## Abstract

The increased demand for genealogical travel to places of family origin in recent years and its consequent economic and social repercussions have justified the aim of this research to obtain the sources of information used by genealogical tourists to prepare for their trip, and the influence that genealogical associations and groups operating on the Internet have on them. For this purpose, a survey was carried out among 223 genealogy enthusiasts who participate in or consult these groups and associations. Through a descriptive statistical analysis of the data obtained, using frequencies and percentages for qualitative variables and means with standard deviations for quantitative variables, this work confirms the outstanding influence that social networks and genealogical associations and groups have on general family history research information, and on specific questions of tourist interest that genealogists receive, and the lesser influence of travel agencies and traditional media. On the other hand, their results support the idea that the main reasons for these tourists to travel are to visit places where their ancestors lived and to research their family history in public archives. All these issues have implications for public cultural and tourism administrations and private sector companies in promoting the provision of historical documentary resources and materials on the internet, as well as the activity of genealogical associations and groups.

## Teaser

Genealogical tourism; Tourist promotion; Sources of tourist information; Social networks; Associations.

## 1 Introduction

Since the second half of the 20th century, tourism has become a fundamental form of human leisure (Richards, 2020), which in the particular case of genealogy enthusiasts is connected to their love of studying and researching their family history (Ong et al., 2017), making the direct search for new family data in existing public or private archives in the places of origin of their ancestors compatible (Mehtiyeva and Prince, 2020); with visiting and living in the natural and urban heritage spaces of these places (Zou et al., 2021), or hiring tourist services and resources (Maleki and Gholamian, 2020) such as accommodation, restaurants, shops, museums or guide services.

In this way, the business tourism sector and the public administrations of the destinations are faced with a type of tourist whose socio-economic profile they need to know, the patterns they follow when travelling and the sources of information they use to plan their tourist trip. These sources of information include general sources such as travel agencies, social networks, references from family members or specialised travel websites, as well as specific sources such as genealogical websites or genealogical groups operating on the Internet.

Up to now, and as will be developed in the first section of this work, research studies have been carried out which have been able to find out the motivation followed by the so-called genealogical tourist when choosing and planning their tourist trip, these include family and cultural motivations (McCain and Ray, 2003). However, there is a lesser degree of knowledge of the means and sources of information from which they obtain the fundamental data to be able to plan and carry out their trip, in addition to general genealogical information, with a special interest in those more specific to the genealogical field, such as genealogical groups and associations that operate on the Internet, as well as the social networks of which they form part.

Considering the aforementioned, this work aims – through the opinion of genealogy enthusiasts themselves who have a relationship with genealogical groups and associations – to contribute to obtaining a better knowledge of these variables, as well as establishing the determinants of the satisfaction with regards to the received information for planning this type of tourism. Consequently, that is the research question that this piece of work aims to answer. Thus, the results which should provide public administrations, and the tourism sector in general, with information to adapt their offer and optimise their promotional resources towards those areas and groups that have the greatest influence on this type of tourist.

The structure of the work begins with a first section describing genealogy, genealogical tourism, and the motivations that various studies have been able to discover about the behaviour of genealogical tourists when planning and carrying out their trips. In addition, it indicates the lack of statistical data that exist for this type of tourism, as well as the best practices developed for its promotion in some countries in the world, and the potential that Spain has for its development. The second section describes the information possibilities offered by the Internet for genealogical tourists. On the one hand, it provides them with availability and access to databases, and on the other hand, it makes it easier for them to exchange experiences and communicate with other genealogy tourists through social networks and genealogical associations. The third section refers to the working methodology which, starting with a survey aimed at members of genealogical associations, and applying a descriptive statistical analysis of the data obtained, using frequencies and percentages for qualitative variables and means with standard deviations for quantitative variables, obtains information on the degree of satisfaction of these people about these associations, and finally the sources of tourist information that they consider most reliable.

The fourth section of conclusions shows that, despite the lack of direct personal relationships, genealogical groups and associations that operate on the Internet have a great influence on genealogical knowledge and relationships between genealogists. Thus, in addition to discovering that finding out about the places of origin of their ancestors and consulting archives are the two main motivations for them to travel, it was found that the most valued and used sources of tourist information are the social networks in which they participate and the recommendations of genealogical groups and associations. All these issues have social implications, including the need to carry out public policies that connect genealogy with the country's culture, greater availability of material resources for people interested in learning about their family history, the establishment of collaboration agreements with genealogical associations to develop activities, and finally the promotion of the public and private sector in group and associative activities through the Internet.

## 2 Genealogical tourism: drivers and current presence

### 2.1 Genealogical tourism and its motivations

The study of the family origin of human beings (Basu, 2004) dates to the first developed forms of human civilisation, mainly as a way of justifying social predominance through the presentation of a supposed mythical past (De Salazar y Acha, 2006). In Spain, it had its first impulse during the 16th to 18th centuries due to the need for social prestige of the noble classes, which was soon imitated by the new bourgeois sectors, and the Jewish and Muslim minorities converted to Catholicism, who used it as a form of integration into the social structure (Soria Mesa, 2004).

Nowadays, this view has been overtaken by a greater popularisation of genealogical practice, with family history research standing out in its general scope, in which historical documentary sources allow the reconstruction of biographical narratives of relatives and ancestors (Higginbotham, 2012), an activity that ends up influencing the research of human identities, individual or collective (Otoo et al., 2021; Salimjan, 2021), carried out in many cases during holiday periods (Hausmann and Weuster, 2018). This practice of holiday travel for the purpose of family history research has various names, such as roots tourism (Mensah, 2015), family history tourism (Santos and Yan, 2010), or genealogical tourism (Marschall, 2015). Although this paper has opted for the latter term since it describes its purpose with a more defined goal, namely a form of tourism in which the people who practice it go to the land of their ancestors (Murdy et al., 2018; Dillette, 2021).

Thus, their own research work is of great importance when it comes to determining the profile of the genealogical tourist, as they consider themselves to be family history researchers, and to a lesser extent mere leisure tourists (Tottola, 2017), a circumstance that has an impact on the objectives they set for themselves when making their tourist trip. Thus, the first of these objectives is to have the opportunity to get to know the present and past social reality in the places from which their ancestors departed (Zou et al., 2021), where the authenticity of the trip will depend on all the memories, cultural discoveries and genealogical information obtained over time (Prince, 2021). This personal perspective makes them travel independently, being able to visit unique places that they consider of personal interest, such as museums, historic buildings, or properties where an ancestor had lived (Marschall, 2015). Among the destinations, rural areas stand out, where these tourists can connect with current local traditions, culture, and daily life (Mehtiyeva and Prince, 2020), in geographical spaces that historically, and still today, suffered from the phenomenon of emigration and depopulation, and therefore in need of tourism activities that can revalue their economy (Quezada-Sarmiento et al., 2018).

Secondly, as researchers, they spend part of their time in the tourist destination consulting archives, libraries, and historical spaces (Santos and Yan, 2010), in order to incorporate new details and family members into their family tree (Pelliccia, 2018). This research work makes them carry out an exhaustive work of collecting and analysing genealogical, historical and tourist attraction information of the place they decide to visit, before, during and after the visit (Mehtiyeva and Prince, 2020), an activity that conditions their tourist experience (Alexander et al., 2017), and which includes religious, military, judicial, legal and civil records (Higginbotham, 2012), which in recent years have been digitised to facilitate public access to face-to-face or digital historical records (Alexander et al., 2017).

In any case, both motivations, the search for identity and research, mean that this type of tourist is not receptive to a generalist tourist offer (Li et al., 2019) and requires specific genealogical services, such as those provided by genealogists, cultural associations and archives, or the organisation of family reunions (Bryce et al., 2017).

Based on the knowledge of the genealogical tourists’ main motivations and patterns, it is worth asking what significance this may have within the general tourism sector. For this reason, we will now analyse the economic data available on this tourist segment, as well as the experience of its activity in some countries around the world, and the potential it may have in the Hispanic cultural sphere.

### 2.2 Genealogical tourism in the world. Perspectives for the hispanic cultural sphere

The first difficulty encountered by any research work on genealogical tourism is the absence of economic data that would allow to illustrate the phenomenon, such as, for example, the number of tourists who practice it, the number of overnight stays, or expenditure on accommodation and food. However, any estimate must be based on its inclusion within Heritage Tourism (Gilli et al., 2013), which in turn is considered a type of cultural tourism, and which means that any analysis can only approach the phenomenon by comparing the behaviour and habits of genealogical tourists with those of heritage tourists (Otoo et al., 2021), or cultural tourists.

Although in Spain, genealogical tourism is currently an underdeveloped niche of the cultural and heritage tourism market, the existence of countries and nations where greater efforts have been made to promote this tourism allows us to analyse and compare the situations and conditions in these places with what could potentially occur in the Hispanic cultural sphere. Of all the receiving markets for genealogical tourism trips, three world geographic areas stand out: Western Europe, West Africa, and the Middle East, mainly Israel; and it is these three geographic areas that have the main issuing markets: the United States and Western Europe, as will be seen below.

With regard to Europe, there are countries that have a certain tradition of caring for this type of tourism. These countries have in common that they have undergone a long historical experience of migratory movements to other states and continents, especially to countries such as the United States, Canada, Australia, and New Zealand (Murdy et al., 2018), where the existing level of economic development has allowed their descendants to have sufficient socio-economic resources to be interested in cultural issues and to make a long-distance journey (Mensah, 2015).

The first of these, Scotland, has developed a marketing brand and tourism strategies around developing its tourism product for people of Scottish ancestry (Bhandari, 2016) estimated at 50 million people, with 455,000 genealogical visits recorded between 2004 and 2014 (Tottola, 2017). The marketing strategy for this type of tourism is centred on the internet (Higginbotham, 2012), with the “VisitScotland” campaign, which generated 25 million visits to its website in its first year of promotion, standing out in recent years (Li et al., 2019).

The second most active European country in promoting genealogical tourism is Ireland, which has 70 million people in the world who are descendants from Irish nationals, having received a total of 830,000 family history tourists between 2003 and 2013 (Tottola, 2017). Within its promotional activities, the so-called Irish Genealogical Project deserves attention, consisting on installing historical database consultation devices with religious and civil references in different parts of the country for general and tourist use (Santos and Yan, 2010).

These two countries were the forerunners in the institutional promotion of genealogical tourism, serving as an example for other countries and states in the world. On the European continent, Germany decided in 2009 to target the US market, as more than 50 million of its inhabitants were of German origin (Marschall, 2015). But this impulse towards the promotion of genealogical tourism also relaunched the outflow of German tourists to Eastern European countries where large populations of German nationality lived before the Second World War, confirming the opportunities of this tourism segment in the country (Marschall, 2015).

Another geographical area with a prominent presence of genealogical tourism in its cultural tourism offer is the West African coast, specifically in countries such as Ghana, Senegal or Benin, where promotional campaigns and activities are carried out for North American tourists of African origin, in which visits to local communities, the recreation of the slave trade and ceremonies and interactions with the local population are planned (Mensah, 2015), with the added objective of carrying out civic engagement work for equality and social justice towards traditionally marginalised groups (Dillette, 2021).

Finally, the reaffirmation of self-identity and legitimisation of traditionally marginalised groups (Ochoa Provoste, 2020), has guided visits by Jewish Americans to Israel and other European states (Sasson et al., 2011), which have included organised activities to religious and family monuments (Li et al., 2019). Within visits to the state of Israel itself, the promotional work of the private company Birthright Israel, promoted by private foundations and the state itself, founded in 1999, stands out, since has allowed up to 750,000 young Jews from all over the world visiting the country (Birthright Israel, 2021): But as in Germany, the promotion of this type of tourism has produced an interest in travel by Israelis to European countries where there were once significant Jewish minorities, demonstrating the circular and lively nature of this type of tourism (Lützen, 2019).

It can be noticed that many of the previous researches refer to the genealogical tourism promotion work done in some countries (Bhandari, 2016; Higginbotham, 2012; Marschall, 2015; Li et al., 2019), but none of them delves into the sources of information that influence the travel decision making of the genealogical tourist, which constitutes a research gap, which was considered relevant enough to justify this work.

Given this background, it is worth analysing the existing conditions in Spain and in those countries with which it has cultural affinities and historical links, in order to be able to analyse whether the realities described above can be extrapolated. Firstly, Spain has been no stranger to the existence of significant population movements throughout its history for economic or political reasons (Garcia de Cortázar, 2003), as is the case in other European countries. These migratory processes have sometimes been directed outside its borders, mainly in America from the 16th to the 20th century (Sánchez-Albornoz, 1990), and in Western Europe during the 20th century (Muñoz Sánchez, 2014), but also internally, from rural to urban areas from the 19th century to the present day (National Institute of Statistics, 2020). These movements have determined that there are currently 2,600,000 people with Spanish nationality in the rest of the world, of which 1,500,000 are in America and 950,000 in the rest of Europe. In addition, there are 483 million people whose native language is Spanish, including countries in Africa and America that were once part of the Spanish colonial sphere (Instituto Cervantes, 2019), as well as thousands of descendants from Sephardic Jews or “Andalusies” Mudejar. All this indicates the existence of a very large worldwide potential market of people who may be interested in learning about the culture and society of ancestors who came from the Iberian Peninsula.

On the other hand, the cultural tourism segment, which includes heritage tourism, has traditional importance in the Spanish tourism sector, which in addition to providing the country with economic profitability, has allowed it to develop in recent years public policies for the preservation of its cultural heritage (Canoves et al., 2016). As can be seen in Table 1, in recent years this importance has been increasing, such that the percentage of foreign tourists whose main leisure activity in Spain is cultural activity has risen from 10.5% in 2015 to 17.3% in 2019, with the number of tourists visiting Spain for this reason increasing by 102.52% in the same period, while their spending grew by 90.8%. Figures for 2020 have not been included as they are considered insignificant due to the effects of the Covid 19 pandemic (Ministry of Culture, 2019).

**Table 1:** Number of foreign tourists and level of expenditure for cultural reasons in Spain from 2015 to 2019.

	2015	2016	2017	2018	2019
Number of visitors (thousands)	7142.8	8014.2	12844.6	12597.8	14465.6
Expenditure (million euros)	8044.3	8567.1	13923.6	13341.1	15348.0
Cultural tourists (%)	10.5	10.6	15.7	15.2	17.3
Cultural tourists expenditure (%)	11.3	11.1	16.0	14.9	16.6
Average tourist expenditure	1016	1028	1063	1084	1101
Average cultural tourist expenditure	1126	1069	1084	1059	1061

Source: Ministerio de Cultura y Deportes de España, Instituto Nacional de Estadística (2021) and Instituto Nacional de Estadística (2016), own elaboration.

Table 1 also shows that there are no significant differences between the ratio of the number of foreign cultural tourists to the total number of foreign tourists and the ratio of foreign cultural tourism expenditure to total foreign expenditure. This similarity could indicate the likeness of the spending habits of this tourist compared to the average foreign tourist, an issue also visible in the average expenditure per person, where figures are again close.

However, knowledge of the motivations of this type of tourist, as well as the experience of genealogical tourism in some countries of the world and its potential for the Spanish tourist market, must be completed with knowledge of the factors which have led to the increase in genealogical practice and the demand for genealogical tourism in recent years. These factors, as will be seen below, are related to the development of new communication technologies through the Internet, and the participation of genealogical tourists in groups and associations that carry out their activity on the global network.

## 3 Sources of genealogical information

### 3.1 Databases and availability of access to information and documents

The emergence of the internet and the wide range of uses it allows has been a fundamental element in developing the genealogy hobby in the last decade (Kennedy-Eden and Gretzel, 2021). It is now possible to carry out research tasks from the homes of genealogists that were previously unthinkable, such as direct access to documentary collections from all over the world (Kramer, 2011); requesting documentation from archives; carrying out genealogical DNA tests (Prince, 2021), and participating in generalist, or thematic and local groups and associations.

Spain has not been a stranger to the process of digitisation of records and documents, and their availability to the public is offered on the websites of various public and private entities, which can be included in the following categories:

• Public administrations such as town councils, regional and state entities. Of all of them, the Portal de Archivos Españoles (PARES) stands out, which brings together the collections of all state archives, including nobiliary, religious, military, judicial, civil service, colonisation of America or Historical Memory after the Civil War, to name the most important (Álvarez-Coca González, 2010). Currently, they have available more than 5 million documents and 35 million images and digitised objects.

From 2015 to 2019 there has been an increase in consultations through the PARES website by almost 50%, a similar increase in all the magnitudes related to the research exercise on the network (Subdirección General de Archivos Estatales, 2019). In addition, tourist visits to the Archive itself have increased by 49.78% in recent years, indicating a greater interest of visitors to Madrid in documentary and archival matters (Subdirección General de Archivos Estatales, 2019).

• Religious, in this case, the Catholic Church, which has documents related to religious sacraments since the 15th century, is the protagonist. Several dioceses that offer access on their websites to indexes of birth and marriage records, from which detailed reproductions can be requested.

In this field, it is also noteworthy the website FamilySearch.org, which belongs to the Church of Jesus Christ of Latter-day Saints. Since 1999, thanks to agreements with some dioceses of the Catholic Church and public institutions, it has thousands of records from all over Spain, available at different levels of access, some immediately for users from anywhere, and others only available in person at their family history centres (Santos and Yan, 2010).

• Genealogical service companies. These companies offer either information services provided by their users but on a paid basis in some of their services (Alexander et al., 2017), with millions of records, such as Ancestry.com, Geneanet.com or MyHeritage.com; or companies that carry out genealogical research on request, including consultancy work and data analysis, as is the case in Spain of tataranietos.com.

We should not forget in this area the services offered by companies that perform genealogical DNA tests, such as Ancestry, Family Tree DNA, Igenea, MyHeritage and 23andMe. In addition to analysing the client's DNA, these companies match the client's DNA with profiles of other people who have also been tested, establishing a network of individuals with a close family match (Fehler, 2011).

The possibility of making prior consultations of genealogical records on the Internet has increased the possibilities of making trips to consult archives in person, as it provides greater prior knowledge of the documents to be consulted without the need for direct advice in the archives (Álvarez-Coca González, 2010), as well as saving time and resources (Longmore, 2000).

But in addition to obtaining this information individually, it is possible to establish social ties with other people who share the same interests and hobbies, and who collectively exert a great influence directly or indirectly on people, their attitudes and behaviour (Kotler and Keller, 2015). Hence the importance of knowing the activity of social networks, as well as groups and associations with a presence on the Internet, which can provide not only strictly genealogical information but also other information that can be used to interact with other people with the same concerns, and to design a genealogical journey, as will be seen below.

### 3.2 Social interactions: social networks and genealogical groups and associations

The internet is not only used for historical research but also for planning and setting up travel projects. For this reason, it is useful to know which tools can be used to obtain information with which to plan and organise a genealogical tourism trip.

Firstly, social networks could be cited as communication tools capable of facilitating the dissemination of content and images, communicating and linking users from different places without the intervention of a professional intermediary (Wong et al., 2019), and managing to transfer opinions and experiences in a continuous and dynamic feedback process (Kennedy-Eden and Gretzel, 2021). As the influence of traditional media has declined in recent years (Nunkoo et al., 2020), social networks have become increasingly important, thanks, among other things, to their ability to create meaning out of information and personal experiences perceived in a spontaneous and disinterested manner (Mehmet and Simmons, 2018), becoming spaces in which public discourses on community issues are generated and circulated (Kou et al., 2017).

The importance of the use of these tools through the internet is somehow traditional in the field of tourism research. Thus, there are studies such as that of Nunkoo, Gursoy and Dwivedi in 2020 that have made it possible to determine the influence that content published on social networks has on changes in the attitudes and behaviour of their users. These are based on theoretical support such as the elaboration likelihood model referring to the influence of information from scientific or socially prestigious sources, the influence of presumed influence model, where the communicative activity of other people is considered, or the social exchange theory on the evaluation of the positive or negative orientation of tourism (Kang and Lee, 2018).

Social exchange theory is concerned with understanding the exchange of resources between individuals and groups in a situation of interaction (Kang and Lee, 2018), an issue of interest in this research because it allows us to theoretically locate social interactions in social networks in which associations and genealogical groups participate. In this way, it can be justified that in these associations there are exchanges of information and opinions with intangible rewards for their members, such as affection (Chang, 2021), respect, competence and effectiveness, and recognition by others (Coulson et al., 2014), or self-development and self-improvement (Paraskevaidis and Andriotis, 2017), without producing, on the contrary, economic counterparts (Coulson et al., 2014). These intangible rewards can also create a personal benefit for each member, or an improvement for the associative collective through greater participation and transmission of knowledge, advice and recommendations in an altruistic manner, in a process facilitated in any case by the existence of social integration rules (Paraskevaidis and Andriotis, 2017) that facilitate implicit or reciprocal negotiations (Coulson et al., 2014).

Furthermore, from the point of view of technology use within tourism expenditure, studies as Do et al. (2020) use the Technology Acceptance Model. Their aim is to confirm that if the usefulness, ease of use and interactivity of mobile phone applications increase, so does the perceived enjoyment and satisfaction of the user and, by extension, the use of these technologies.

On the other hand, social networks strongly influence destination choice, especially when tourists regularly participate in these networks, when they have less knowledge about their destination, and when trip planning requires greater complexity and difficulty (Tham et al., 2020), all of which are relevant in genealogical tourism, as genealogical tourism requires specific historical and cultural knowledge on the subject. In any case, the tourist's trustworthiness influences the choice of tourist destination, as the opinion, behaviour and decisions of certain people are considered decisive in making a travel decision (Berhanu and Raj, 2020), the most relevant being those received by family and friends; and then the recommendations, comments and information obtained on social networks (Pabel and Prideaux, 2016). All these issues can be reflected in the recommendations and consultations made to qualified people from genealogical groups and associations.

For all these reasons, there are precedents of tourist destinations developing their campaigns on international social networks, in some cases by hiring well-known bloggers (Avraham, 2015), in others by encouraging travellers to share their impressions of the trip (Dillette, 2021). In this way, tourists make use of social networks such as Facebook, WhatsApp, YouTube, Twitter or Instagram to share their travel experiences and opinions (Timothy 2018), which ends up influencing the decision-making of other tourists with similar tastes and receptiveness towards the content of these experiences and opinions (Cuomo et al., 2021). In this way, the number of tourists interested in this type of cultural heritage tourism expands, as the lack of cultural and historical knowledge is compensated by the possibilities offered by technology to provide accessible information to all audiences, improving their experience at the destination before starting the trip (Hausmann and Schuhbauer 2020).

Secondly, the existence and activity of genealogical associations and groups operating on the Internet are also facilitating the needs of genealogical tourists to obtain pre-travel references, including receiving information, advice and the possibility of interaction with other people as happens in other business and educational sectors, which ultimately enhances community development and the building of the association's social capital (Zalon, 2008).

Thus, the theoretical debate on the social activity of members of an association through the internet ends up deriving in what are the reasons that make people decide to belong to these associations, and the personal and social benefits they obtain from it. There is a wide literature in different fields of study that has been interested in the mutual influence that is established between associations and their members. Thus, based on Private and Public Engagement Motivation Theories and referring to the nonprofit association sector, it has been argued that there are two main types of motives for individuals to participate in these collective action associations, on the one hand, to benefit themselves, and on the other to benefit the common good (Young and Berlan 2021). This idea underpins that there are two personal motivations for association membership and that serve as a theoretical basis for justifying the search for social trust and access to the collective resources of these associations (Lanero et al., 2017). Firstly, the so-called instrumental motivations, aimed at helping other members of a community of equals and therefore incentivising collective and social interests (Valeau et al., 2019), which in this case may correspond to the need to give indications and advice to other members on how to search for genealogical information, the existence of common ancestors, interpretation of ancient documents and places to visit at the destination. Secondly, expressive motivations, aimed at satisfying the member's private interests thanks to the desire to socialise with people with whom they share affinities and hobbies, which in the case of members of genealogical associations and groups corresponds to the personal relationship at a distance with other people in the collective (Valeau et al., 2019).

In addition, genealogical associations and groups also become spheres of socialisation, as they are made up of people who decide coming together because of shared interests and aiming to transmit and receive knowledge (Hager, 2014), but with different characteristics from one another.

The former is made up of people who decide to come together because of their interest in genealogy, transmitting information and carrying out a didactic task of promoting genealogical work and maintaining collective identities by means of a formal and legally recognised structure (Pelliccia 2018). Some of these associations provide databases and collaboration to locate ancestors (Li et al., 2019), thus managing to maintain ties between people outside their family destinations, and preserving cultural elements, something that stands out in young people with higher educational levels (Tottola, 2017).

Thanks to this collaborative work, the sociability of their members is enhanced, exchanging information about their respective experiences and advice on how to carry out their research (Legrand, 2002). As is the case in other associative sectors (Lovejoy and Saxton, 2012), these associations focus their activity on the Internet, which is why most of them started their activity from 2000 onwards. That facilitates the relationship between people of different geographical origins, age, or economic position, producing an interrelationship between their members that is much greater than that which could exist in person, as the geographical and time limitations of any face-to-face activity can be avoided.

In the case of Spain, the associations dedicated to genealogy can be classified into two categories:

On the one hand, those with an institutional character as the Real Academia Matritense de Heráldica y Genealogía, which is Spain's representative in international genealogy and heraldry bodies. Its main activity is research and dissemination in these fields, for which it produces publications, conferences and reports for official bodies and cultural entities, as well as being present on social networks. This type of association stands out for its activity in scientific promotion and dissemination, but without establishing day-to-day relations between its members.

On the other hand, there is a second type of association with a less institutional character and aimed at a public whose operating philosophy is based on altruistic cooperation, sharing the work of family genealogical research and advice among its members to ultimately achieve an atmosphere of community among equals. The first association created in Spain with these objectives was the Asociación de Genealogía Hispana (Hispagen), which brings together genealogy enthusiasts from all over Spain and Latin America. This association makes available to its members several genealogical databases of some localities, genealogical trees of members, as well as a consultation area between members to share discoveries with the community.

From this national association, different associations of a more local nature have arisen throughout the country, with the same general and popular essences, such as Antzinako, which was set up in 2005 and brings together amateur genealogists interested in researching family origins in the Basque Country and Navarre. In its beginnings, it imitated the operating patterns of the family history associations of the French Basque Country, to which they were united by cultural affinities and geographical proximity, presenting a wide range of activities to help research and promote genealogical family history through its website antzinako.org, among which are included:

- Extensive database with historical documents and family trees of members.
- Links with public and private institutions.
- Publication of a biannual online magazine called Antzina.

Another area in which it stands out is the work of dissemination and collaboration in genealogical research. In this case, this work is not carried out directly on the website but by using a group related to the Association on the Google Groups platform provided for this purpose by Google. Daily, questions and answers are generated from members about doubts regarding data on locations, management of procedures and timetables of archives, the origin of surnames, localities, interpretation of documents, or coincidence of relatives, mainly.

There are other active associations in Spain, such as the Asociación Cultural de Genealogía e Historia de Aragón, the Asociación Cántabra de Genealogía, the Asociación Canaria de Genealogía e Historia Familiar, the Asociación Riojana de Genealogía y Heráldica, in Galicia, the Asociación Xenealoxia.org, the Asociación Raíces Reino de Valencia, which also operates in a similar way to Antzinako.

Finally, reference should be made to genealogical groups, which are made up of people who share their love of genealogy, also using the Internet as a tool for communication and interrelation between members, but without creating a formal associative structure. Therefore, they have no official rules of operation, nor management positions (at most administrators of Internet groups), using the service provided on the Internet by Google, Google Groups. These groups differ from associations in that the former tend to focus on creating community and cooperation, providing information permanently on their website, while the groups share their discoveries in a more informal way, sharing with associations the geographic character as a constitutive element of the same.

In spite of all this, these groups carry out a work of promotion and collaboration for genealogical research, and some of them have been monitored, such as Genealogía Andalucía Occidental, Genealogía Andalucía Oriental, Genealogía de México, Genealogía de Puerto Rico, Linajes Malagueños and Genealogía Soria, where we can see mainly a work of consultation and advice; and in the last two, an offer of information on their web pages with questions related to genealogical trees, heraldry and recommendations for research in historical archives.

From this background, this work proposed the need to know the influence that these groups have among people who go on genealogical trips through the direct experiences and opinions of the latter, considered the target audience of the study, as well as to compare this influence with that of other more traditional means of tourist information such as that provided by travel agencies and traditional media. In this way, it was set as a goal to determine the most satisfactory channels through which genealogical travellers develop their trips and specifically the role genealogical groups and associations have in transmitting valid information to prepare the tourist trip, and general genealogical information.

## 4 Methods

### 4.1 Survey

A questionnaire (Supplementary Table 1) was carried out to collect socio-economic aspects, degree of commitment, participation in genealogical groups and evaluate the resources used to plan a tourist journey. For the elaboration of the questionnaire, an exhaustive bibliographic review of the field of study was carried out in publications indexed in academic repositories such as Web of Science and Scopus, including terms such as genealogical tourism, cultural and heritage tourism, motivation, and genealogical associations.

From this bibliographical review, it could be seen, in agreement with Zhu and Airey in 2021, that most of the research works on this subject based their methodology on qualitative techniques. Consequently, the three blocks that made up the questionnaire were determined from the few research works on genealogical tourism that used quantitative methods with surveys and from others in the generic field of cultural tourism or non-profit associations.

The first block of seven questions refers to the socio-economic data of the sample, for which the work of Otoo, Kim, and Choi in 2021 was used to collect data related to gender, geographical origin, and socio-economic status of a sample, which was used to find out about the tourists' motivation diaspora. The second block of five questions was based on the work of Kozak in 2002. More specifically, in aspects referring to the degree of motivation to undertake tourist trips, as well as the degree of satisfaction with the objectives set for this type of trip. These type of questions and their measurement were appropriate in this research work to analyse the extent, motives, and goals set, as well as the degree of satisfaction with these objectives in the genealogical trips of the sample. The third and last block of five questions asked about the degree of affiliation, motivation, and satisfaction of the respondents with a genealogical association, as well as their favourite sources of information for preparing a genealogical trip. In this case, it was necessary to turn to research from outside the field of tourism, namely Ki & Cho's work in 2021 on non-profit association members' supportive behaviours, which used for its analysis a questionnaire assessing, among other things, the personal benefits, and professional benefits of belonging to a non-profit association.

From the information obtained in the bibliographic review, a first draft of the questionnaire was designed. The final draft was validated with the opinion of 3 members of an association or genealogical group in Spain, with an overview of the subject under study, presenting the main blocks into which it was to be divided and the questions included in each one. As a result of this first review, contact with distant relatives was included as an option in the motivations for the genealogical journey.

Finally, the questionnaire was designed in Spanish for all the Spanish and Latin American groups and associations, and also translated to English for those in the United States. The items were evaluated on a 10-point Likert scale (0 strongly disagree, and 10 strongly agree) (Otoo et al., 2021), except for sociodemographic variables and those related to numbers and reasons for travel, for which categorical data were used. It was prepared and sent using the Google Forms web application (October–December 2020), with the collaboration of the administrators of the main genealogical groups and associations in Spain and Latin America with Spanish family ties and origins so that it could be distributed among their associates and members (Supplementary Table 2), a list of which is included in an attached appendix. The subsequent distribution among the members was diverse. Most sent the questionnaires to the internal forums of the association or group, appearing in Google Groups chats or sent to e-mails, while only one of them chose to publish them on the social networks where the association participates.

These questionnaires analysed variables that are described in the following Table 2:

**Table 2:** Description of the variables used in the analyses.

Variables	Categories
Sociodemographic	
Age	In years and from categories: 18–34/35–48/49–64/65 years or more.
Gender	Male/Female
Marital status	Single/Married or in a relationship/Separated/Divorced/Widowed
Employment Status	Employed/Unemployed/On sick leave/Retired or early retired/Student/Housework
Travels	
Travel in the last 3 years	Yes/No
Genealogical tourism journey	Yes/No
Other reasons for travel	Tourism and leisure/Work
Reasons for genealogical travel	Consultation of archives/Visiting places where ancestors lived/Meeting family members
Degree of satisfaction with the objectives of the genealogical journey (0–10)	Consultation of archives/Visiting places where ancestors lived/Meeting relatives/Money spent on trips
Money spent on travel	In euros
Degree of satisfaction with the support provided according to the information source (0–10)	Family and friends/Travel agency/Media/Genealogical associations/Social networks
Genealogical association	
Membership in a genealogical association	Yes/No
Reasons for attending the genealogical association	Origin of surnames and heraldry/Existence of ancestors shared with other people/Information from archives/Geographical locations/Contact with distant relatives/Learning to investigate
Evaluation of the support provided (0–10)	Origin of surnames and heraldry/Existence of ancestors shared with other people/Information from archives/Geographical locations/Contact with distant relatives/Learning to investigate

Source: Own elaboration.

### 4.2 Sample

Regarding the selection of the sample, this study considered that the need to have a representation of people who will potentially undertake tourism trips of a genealogical nature could only be guaranteed by going to associations and groups where these types of tourists interact regularly. Moreover, it was known from previous interviews that the associations and groups had an average membership of between 100 and 300 members, with the added problem that there was a lack of official statistics that could help determining a reliable sampling frame. For this reason, a convenience sampling technique used in diaspora tourism studies, such as that of Otoo, Kim and King in 2021, was chosen as more appropriate for surveying an audience whose common characteristic was membership in genealogical groups and associations. For this reason, the survey used is of a cross-sectional type, sent indirectly to participants randomly without selecting a specific profile, through the group participation bodies of the associations themselves, in such a way that some of them sent them in their participation forums, others sent them by e-mail and others shared them on their social networks. We obtained a sample of 223 participants, with an average age of 59 years, of whom 51.1% were between 49 and 64 years old, 68.0% were married or in a couple, and 39.5% were retired. We found a sample without gender bias (53.4% men vs. 46.6% women), where the sample size obtained was considered sufficient to obtain conclusive evidence from the statistical analyses carried out. In addition, it should be considered that genealogical tourists are a small group of the population of cultural heritage tourists, which means that large samples are not necessary for them to be representative.

**Figure 1:**
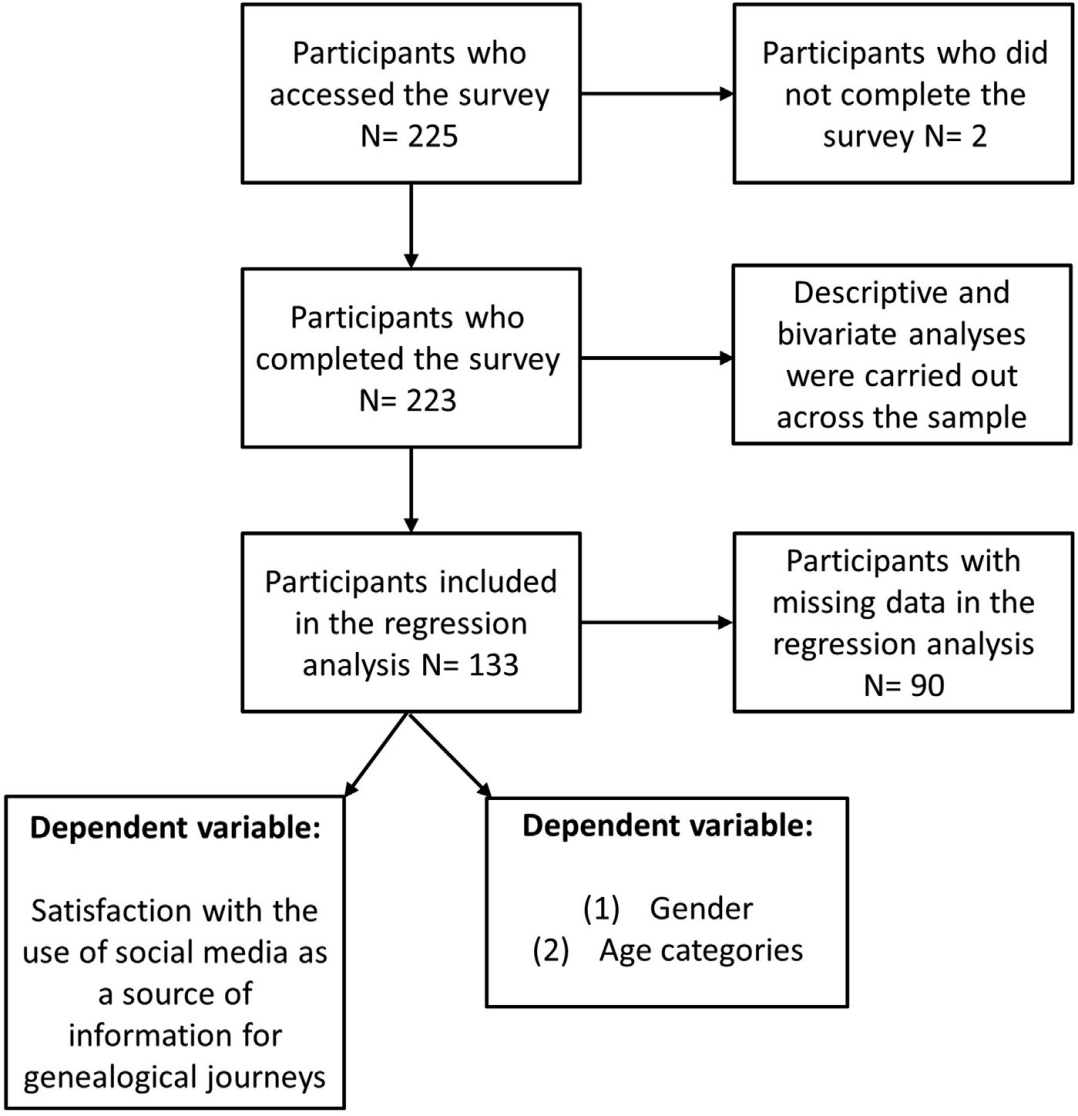
Sample selection flow chart. Source: Own elaboration.

The following flowchart in Figure 1 shows the selection and analysis of the sample.

### 4.3 Statistical analysis

First, after cleaning and sieving the data to obtain a consistent sample, descriptive statistics were performed on all variables to contextualise them in the situation of our respondents. Descriptive analyses were carried out using frequencies and percentages for qualitative variables and means with standard deviations for quantitative variables, which assessed the normality of the variables, resulting in a population that did not have a normal distribution (Table 3), so it was necessary to apply non-parametric statistics.

**Table 3:** Descriptive analysis of the sociodemographic and genealogical characteristics.

		Mean ± SD or (%)	Skewness (Kurtosis)	CI (95%)
Sociodemographic variables				
Age [N: 222]		58.8 ± 11.96	−.668 (1.07)	57.19–60.33
Age intervals [N: 222]	16–34 years	8 (3.6)	−.471 (−1.74)	21.01–30.24
	35–48 years	29 (13.06)	-.393 (−1.14)	41.87–44.68
	49–64 years	116 (52.25)	−.139 (−1.20)	56.59–58.17
	65 years or more	69 (31.08)	.846 (.32)	70.25–72.59
Gender [N: 222]	Men	119 (53.6)	–	–
	Women	103 (46.4)	–	–
Marital status [N: 222]	Single	36 (16.29)	–	–
	Married or in a relationship	149 (67.42)	–	–
	Separated/divorced	28 (12.67)	–	–
	Widow	8 (3.62)	–	–
Employment status [N: 222]	Employed	110 (49.55)	–	–
	Unemployed	7 (3.15)	–	–
	On sick leave	3 (1.35)	–	–
	Retired or early retired	88 (39.64)	–	–
	Students	3 (1.35)	–	–
	Housework	11 (4.95)	–	–
Travels				
Travels in the last 3 years [N: 222]	Yes	212 (95.5)	–	–
	No	10 (4.5)	–	–
Genealogical tourism travel [N: 215]	Yes	140 (65.12)	–	–
	No	75 (34.88)	–	–
Other reasons to travel [N: 121]	Tourism and leisure	104 (85.95)	–	–
	Work	17 (14.05)	–	–
Reasons for genealogical travel				
Consultation of archives [N: 140]		94 (67.14)	–	–
Visiting places where ancestors lived [N: 140]		114 (81.43)	–	–
Meeting relatives [N: 140]		26 (18.57)	–	–
Degree of satisfaction with the objectives of the genealogical journey (0–10)				
Consultation of archives [N: 94]		6.8 ± 3.03	−.721 (−.632)	6.3–7.3
Visiting places where ancestors lived [N: 114]		7.51 ± 2.95	−1.026 (−.314)	7.02–8
Meeting relatives [N: 26]		8.04 ± 2.84	−1.281 (.387)	7.57–8.51
Money spent on travel (total) [N: 122]		2677.54 € ± 2730.80	1.22 (.39)	2192.97–3162.11

Source: Own elaboration.

Within this non-parametric statistic, it was decided to apply the Mann-Whitney test (McKnight and Najab 2010) was used to assess differences in the distributions of quantitative variables (age, money spent on travel and satisfaction with the help provided according to the source of information) with respect to the dependent variable (type of tourism: genealogical/other). This test has precedents in the heritage tourism field to analyse significant differences in a sample on the perception of historical heritage and cultural interest (Pérez-Gálvez et al., 2019) or on the motivational dimensions for visiting a heritage tourism city (López-Guzmán et al., 2019). Besides, the Pearson Chi-Square test was used to compare the distribution of two categorical variables (McHugh, 2013), used in the field of tourism to measure issues such as business pay differences between sexes (Handaragama and Kusakeb, 2021), or in the field of financial investments to measure the relationship between demographic characteristics and decision-making (Lan et al., 2018), has been used to assess differences in the distributions between qualitative variables (sex, marital status, and employment status) and the dependent variable (type of tourism: genealogical/other).

Lastly, multiple linear regression (Uyanık et al., 2013) was used to evaluate the association between the independent variables: male gender (ref. female gender) and the age ranges 18–34, 35–48 and 49–64 (ref. 65 years or older), taking as the dependent variable the degree of satisfaction of using social networks as a source of information for their genealogical travel (0–10). The statistical treatment of the independent variables is the one given to dummy variables, being 1 the studied characteristic and 0 the reference value, which is excluded from the equation to avoid collinearity problems, as usual in this type of regressions. On the other hand, the dependent variable is a continuous variable within the interval 0 to 10, representing the score of the level of satisfaction. That is, it is a quantitative variable, which allows it to be considered as dependent variable in a multiple linear regression. Due to its nature, treating it as an ordinal qualitative variable is dismissed. The estimation of the model resulted in statistically significant results at different levels, but most of them under the 0.05 threshold. The calculations were run using SPSS v. 26.0. Betas, p-values and 95% confidence intervals (CI 95%) were shown.

### 4.4 Results

Regarding the main sociodemographic variables, as can be seen in Table 3, the mean age of the participants was 59.1 years, of which 51.1% were between 49 and 64 years of age, and 68.0% were married or in a couple, and 39.5% were retired.

From the point of view of tourism practice, 95.5% of the participants had made a trip in the last 3 years, of which 67.6% were for genealogical reasons. Of the 67 participants whose motive was not genealogical, we found leisure and tourism (92.4%) or business travel (19.7%).

The most frequent reasons related to genealogy were visiting places where ancestors lived (81.4%), consulting archives (66.4%) or meeting relatives (18.6%). The degree of satisfaction for these reasons (from 0 to 10) was 7.4, 6.2 and 5.2, respectively. Of the different sources of information used by the participants, the most satisfactory were genealogical associations (6.7 out of 10) and social networks (9.1 out of 10). The average amount of money spent on genealogical trips was 2471.9€ (Table 3).

67.6% (n = 140) made genealogical trips, compared to 32.4% (n = 67) who made trips for tourism, leisure or work.

The mean age of both groups was similar at around 59 years (p = 0.817), while, for gender, females had a higher proportion of genealogical travel than for other reasons (49.3% vs 34.3%, p = 0.043). 66.9% of those engaging in genealogical tourism were married or in a relationship (vs 70.1% travelling for another reason), although the largest difference was for those separated/divorced (15.8% vs 3.0% for another reason, p = 0.033).

For employment status, no significant differences were found, although it is worth noting the high proportion of retirees or early retirees who made genealogical travel as well as travel for other reasons. In general, genealogical tourists spent less money on their trips (2366.7€ vs. 3792.6 for another reason, p = 0.061), although these differences were not statistically significant. These results coincide with the existing data for cultural tourism in Spain according to the National Institute of Statistics in 2021, shown in Table 1, which would allow us to affirm that the socioeconomic level of this type of tourist is not very different from that of other types of tourists in the cultural heritage segment (National Institute of Statistics, 2021).

Concerning the degree of satisfaction with the information received, as can be seen in Table 4, genealogical tourists were less satisfied with the information received from family or friends (5.8 vs. 7.4, p = 0.078), travel agencies (2.1 vs. 4.1, p = 0.009), or the media (4.6 vs. 4.7, p = 0.900). However, this satisfaction increased in the case of genealogical groups and associations (6.7 vs. 6.7, p = 0.867), and especially that received from social networks, where the highest rating of all the alternatives presented was reached (6.2 vs. 5.6, p = 0.503). Although regarding the degree of satisfaction, the differences were only significant for the information received from travel agencies (Table 4).

**Table 4:** Bivariate analysis of sociodemographic characteristics, degree of satisfaction with the information received according to the type of tourism undertaken.

	n (%) o mean ± SD		P-value
	Genealogical tourism 140 (67.6%)	Other reason^1^ 67 (32.4%)	
Sociodemographics			
Age	59.7 ± 11.5	59.3 ± 12.1	0.817
Gender			**0.043∗**
Male	71 (50.7)	44 (65.7)	
Female	69 (49.3)	23 (34.3)	
Marital Status			**0.033∗**
Single	20 (14.4)	14 (20.9)	
Married or in a relationship	93 (66.9)	47 (70.1)	
Separated/divorced	22 (15.8)	2 (3.0)	
Widow	4 (2.9)	4 (6.0)	
Employment status			0.120
Employed	65 (46.4)	39 (58.2)	
Unemployed	8 (5.7)	1 (1.5)	
On sick leave	3 (2.1)	0 (0.0)	
Retired or early retired	51 (36.4)	26 (38.8)	
Students	2 (1.4)	0 (0.0)	
Housework	11 (7.9)	1 (1.5)	
Money spent on travel	2.3667 ± 2.9647	3.7926 ± 6.6936	0.061
Degree of satisfaction with the support provided according to source of information			
Family and friends	5.8 ± 3.5	7.4 ± 2.6	0.078
Travel Agency	2.1 ± 3.1	4.1 ± 3.4	**0.009∗**
Media and communication	4.6 ± 3.3	4.7 ± 3.0	0.900
Genealogical associations	6.7 ± 3.2	6.9 ± 2.9	0.867
Social Networking	6.2 ± 3.2	5.6 ± 3.6	0.503

1 Including tourism, work or leisure Source: Own elaboration.

With respect to their degree of relationship with genealogical associations and groups, the data in Table 5 confirm that 92.8% of them belonged to some of them, with a high degree of satisfaction with the information received in their social interactions through the Internet. This satisfaction is evident in the main reasons for going to genealogical associations, with the most important being to learn how to do research (72.9%), the existence of ancestors shared with other people (67.4%) or information from archives (61.2%). Satisfaction with the information received was especially high for those who were helped in consulting archives (7.9 out of 10) and in learning research techniques (7.6 out of 10) (Table 5).

**Table 5:** Membership, reasons for use and value given by genealogical associations.

Genealogical association		
Membership N: 139	Yes	129 (92.8)
	No	10 (7.2)
Reasons for joining the association N: 129	Origin of surnames and heraldry	56 (43.4)
	Existence of shared ancestors with other persons	87 (67.4)
	File information	79 (61.2)
	Geographical locations	42 (32.6)
	Contact with distant relatives	43 (33.3)
	Learning to research	94 (72.9)
Evaluation of the support provided (0–10)	Origin of surnames and heraldry N: 95	6.8 ± 3.0
	Existence of ancestors shared with other people N: 104	6.8 ± 3.0
	Information from archives N: 102	7.9 ± 2.6
	Geographical locations N: 85	7.2 ± 2.8
	Contact with distant relatives N: 82	5.6 ± 3.6
	Learning to investigate N: 110	7.6 ± 3.0

Source: Own elaboration.

From the multiple linear regression analysis shown in Table 6, it was found that males who belonged to genealogical associations had a higher degree of satisfaction with the use of social networks as a source of information for their travels compared to females (B = 2.915, 95% CI 2.320 to 3.511). Furthermore, with respect to older commuters (>64 years), those aged 18–34 years (B = 4.835, 95% CI 1.162 to 8.508) and those aged 49–64 years (B = 2.106, 95% CI 0.884 to 3.329) were associated with a higher degree of satisfaction with the use of social networks as a source of information for their genealogical travels (Table 6).

**Table 6:** Multiple linear regression to analyze factors associated with improved satisfaction with the use of social networks as a source of information for genealogical travel (N = 133).

		Ref.	Multiple linear regression		
			B	p-value	95% CI
Gender	Male	Female	2.915	**<0.001∗**	2.320, 3.511
Age	18–34	>64	4.835	**0.010∗**	1.162, 8.508
	35–48		1.984	0.068	−0.149, 4.118
	49–64		2.106	**0.001∗**	0.884, 3.329

Source: Own elaboration.

## 5 Discusión and conclusión

### 5.1 Discussion of findings

The data and conclusions of this research were obtained from a sample in which 92.8% of the respondents belonged to a genealogical association, had participated or had been informed by a genealogical association. For these, satisfaction with the information received in these genealogical associations was especially high for consulting archives and learning research techniques, which is in line with that observed by Ki & Cho in 2021 in United States engineering association memberships, and by Ebbers, Leenders and Augustijn in 2021, who, among a sample of members and non-members of the visitors' association of the Hermitage Museum in Amsterdam, finding that partnerships are able to provide value creation in their activities, as well as social prestige. In addition, men belonging to genealogical associations and age categories 18–34 and 49–64 were associated with a higher degree of satisfaction using social networks as a source of information for their genealogical journeys. Regarding gender data, there is a variety of results in previous research. On the one hand, some of them obtain results similar to those of this paper (Assaker, 2020), others, however, conclude the opposite (Escobar-Rodríguez and E Carvajal-Trujillo, 2014), and finally, there are some that do not find significant differences in their use between sexes (Berhanu and Raj, 2020). As for those referring to the use of social networks according to age, there are differences with respect to the results obtained in research as that of Berhanu and Raj in 2020, for whom the use of social networks as sources of information for travel and tourism among visitors to different cultural and nature tourism destinations in Ethiopia decreases progressively from the age of 46 onwards, without there being any intermediate scale with greater satisfaction, as occurs in this work.

Particularly remarkable, as well as statistically conclusive, is the low rating given to the information provided by traditional travel agencies and conventional media, which was already pointed out by Nunkoo, Gursoy and Dwivedi in 2020 for the case of residents¨ reactions to tourism development. Respondents are more satisfied with information obtained from family and friends, genealogical associations and groups and social networks (6.2, 6.6 and 6.2, respectively, on a scale of 0–10), results that coincide with the work of Pabel and Prideaux in 2016, and Berhanu and Raj in 2020, although the latter conditioned satisfaction with social network information on it coming from real users and with quality content. In this sense, statistically significant differences were only found for marital status, specifically for separated people, who obtained satisfaction of 8.9 from the information received by genealogical associations (compared to 6.2 for single people, 6.3 for married people and 7.3 for widowed people). Thus, the Internet and the opinions of people without professional ties or direct economic interests in the practice give more satisfactory information than traditional travel companies.

Finally, the search for and discovery of places linked to family roots have been confirmed, in line with other research carried out in other countries and with different research methodology, such as that of Alexander, Bryce and Murdy in 2017, based on in-depth interviews with genealogical tourists, in which the objectives stated by genealogical tourists for their trips coincide. However, and also in line with other research work such as that of Longmore in 2000 for archives available on the internet in the United Kingdom and Ireland, there is a second important motive, and that is the need to go to places that have historical archives to consult, even though the internet also facilitates the search for family historical references. In this case, public or private websites facilitate the task of researching family history and precede the face-to-face search, increasing in practice the number of trips to places of family origin.

### 5.2 Conclusion

This research work makes new contributions to the knowledge about behaviours and patterns of genealogical tourists, specifically those referring to the sources to which they turn for advice on aspects related to genealogical practice and the planning of genealogical tourist trips.

Thus, in the first place, satisfaction with the information they obtain through the altruistic and disinterested collaboration of other people with whom they share hobbies through groups and associations that operate on the Internet stands out. This result coincides with other previous studies, such as Lovejoy & Saxton in 2012, for whom using social networks in non-profit associations creates more intense forms of public participation and the capacity to build a sense of community. This idea links this research to the fundamental aspects of social exchange theory, as intangible and altruistic motivations are the ones that influence participation in these groups and associations. The information that is most demanded and valued in these associations and groups is that related to genealogical research, specifically the transmission of knowledge about research techniques and tips, as well as the sharing of local family lineages and matching ancestors. That is relevant because it takes place in a context in which most of the feedback processes of consultations and responses take place without direct personal contact, between people who are mostly over 50 years old, using remote means through the internet.

Secondly, this study has shown that the majority of genealogy enthusiasts who participate in genealogical groups or associations go on genealogical trips, which is more prominent among those who are older and retired.

Thirdly, it was found that of the possible sources of information that can be used to prepare a genealogical trip, tourists in this segment of cultural heritage tourism especially use and value information obtained from genealogical groups and associations, as well as information from social networks. This conclusion is consistent with other research, such as that of Cuomo, Tortora, Foroudic, Giordano, Festae and Metallo in 2021, which found that social networks provide information quality, credibility, interaction, and accessibility.

Therefore, it can be concluded that this type of tourist, regardless of age, gender and socio-economic status, uses and positively values social relations and the search for genealogical and tourist information via the Internet. This facilitates, generalises and increases genealogical practice, and by extension the possibilities of increasing tourist trips for this reason, which could contribute not only to the development of this particular tourist segment but also to the general strengthening of cultural heritage tourism. This was idea already pointed out by Dillette in 2020 when he concluded that sharing personal experiences on social networks about identity and cultural community, provides a greater propensity to travel to places with family ties.

To achieve this objective, public administrations must recognise the value that general genealogical research has on the promotion of culture and urban and natural heritage, as well as for the development and consolidation of tourist activity in areas of the country, especially rural areas, which suffered, and still suffer, extensive emigration processes. In tourism fields other than genealogy, such as ecotourism, it has been highlighted that tourism promotion in rural areas contributed to improving the environmental awareness of tourists and the local population, as well as to preserve the natural heritage of these areas (Quezada-Sarmiento et al., 2018).

From a cultural point of view, genealogy can become an instrument that promotes the country's image, taking advantage of the interest of many people with ancestors of Spanish origin in learning about the history, customs and art of the place of their family origin. As Etemaddar, Duncan and Tucker stated in 2016, the diaspora tourist not only wishes to visit a place linked to their family origins but also wants to acquire feelings, a sense of belonging and identity. This knowledge would increase the demand for other cultural products, as well as strengthen fraternal relations and cultural exchanges between countries from different continents but with common historical links. This extent is related to the conclusions of Panibratov and Rysakov (2021), for whom the existence of links between people and companies from places that have had diaspora phenomena in origin and destination increases commercial exchanges and tourist visits between them. Hence, public cultural institutions should take advantage of their physical offices abroad and their Internet resources to provide information on archives, associations, and historical and artistic content, with special significance for millions of nationals from different Latin American, European, North African and Israeli countries. That would achieve a promotional activity similar to those existing in other European and African countries. However, as reflected in some works such as Murdy's in 2018, its success has been determined by the purchasing power of the citizens of those countries.

But, in addition, the documentary collection of public and religious archives should be digitised and made accessible on the internet to make them available for genealogical, academic or personal research since, as Prince concluded in 2021, the digitisation of genealogical records popularises genealogy, and increases the possibilities for genealogical travel.

This cultural public policy action is linked to another of the major implications of this work: the importance of collaboration between the public and private sectors, and genealogical associations, which is an idea that has similarities with recommendations in the field of sustainable development of cultural heritage tourism in works such as Asmelash and Kumar in 2019. In our case, we argue that public and private resources for promotion and marketing should be allocated to these associations and groups, which will allow administrations and companies to be present in their activities and recommendations. In this way, agreements and collaborations can be established to promote genealogical activity through the cataloguing and indexing of historical documents, publication of research, organisation of didactic promotional conferences, visits to archives and places of historical interest for members of associations and groups, or advisory work for genealogical tourists via the Internet, which would not only benefit this type of associations but also others dedicated to the promotion of local history or the historical memory of the Spanish Civil War and the dictatorship.

### 5.3 Limitations and future research

The main limitation of our work is that the public under study is not the generality of genealogy enthusiasts who can practice genealogical tourism but those who have participated or are part of a genealogical group or association. That means that there is a part of genealogy enthusiasts whose opinions and preferences are not collected and who, in the future, with other means of research, will have to be analysed in order to obtain more generalisable conclusions.

In addition, future research will have to focus on other variables, including the purchasing power of these tourists, their consumption patterns, the means of transport used to make their trips, the average expenditure at the destination, the types of accommodation and the frequency with which they engage in this type of tourism.

## Declarations

### Author contribution statement

Ricardo Urrestarazu-Capellán: Conceived and designed the experiments; Performed the experiments; Analyzed and interpreted the data; Contributed reagents, materials, analysis tools or data; Wrote the paper.

Jose Correa-Fernández: Performed the experiments; Analyzed and interpreted the data; Contributed reagents, materials, analysis tools or data; Wrote the paper.

Francisco Sánchez-Cubo: Analyzed and interpreted the data; Contributed reagents, materials, analysis tools or data.

### Funding statement

This research did not receive any specific grant from funding agencies in the public, commercial, or not-for profit sectors. Funding for open access charge: Universidad de Málaga / CBUA.

### Data availability statement

Data included in article/supp. material/referenced in article.

### Declaration of interest's statement

The authors declare no conflict of interest.

### Additional information

No additional information is available for this paper.

## Appendix A Supplementary data

SUPPLEMENTARY TABLE 1.docx SUPPLEMENTARY TABLE 1.docx

SUPPLEMENTARY TABLE 2.docx SUPPLEMENTARY TABLE 2.docx
