# Supplementary material for: The sources of information of the genealogical tourist: the influence of social networks and genealogical associations
Source: Heliyon. 2022 Nov 10;8(11):e11551. doi: 10.1016/j.heliyon.2022.e11551 (PMC9668519; doi:10.1016/j.heliyon.2022.e11551)
Supplement: SUPPLEMENTARY TABLE 1.docx [file mmc1.docx]

Appendix 2. Questionnaire

1. Datos sociodemográficos:
2. Por favor, indique su año de nacimiento
3. Por favor indique su género:

- Mujer
- Hombre
- Otra

1. Por favor indique su país de residencia
2. Por favor, indique su nacionalidad:
3. Por favor, indique su estado civil:

- Soltero/a
- Casada/o o en pareja
- Separado/a o divorciada/o
- Viudo/a

6. Por favor, indique su situación laboral actual:

- Trabajando
- En desempleo
- Jubilada/prejubilada
- De baja laboral
- Ama de casa
- Estudiante

1. Turismo genealógico.

7. ¿Ha realizado algún viaje en los últimos 3 años? *

- Sí
- No

8. ¿Alguno o algunos de ellos era un viaje de Turismo genealógico?

- Sí (Marque y pase a la respuesta 10)
- No (Marque y pase a la respuesta 9)

9. Entonces, ¿Cuál era los objetivos de su viaje? (Marque y pase a la pregunta 17)

- Turismo y ocio
- Trabajo

10. ¿Cuáles eran los objetivos de su viaje genealógico?:

- Consultar archivos
- Visitar lugares donde vivían mis antepasados
- Conocer familiares

11. Indique el grado de satisfacción sobre los objetivos de su viaje (siendo 0 el peor grado y 10 el máximo grado de satisfacción)

0 1 2 3 4 5 6 7 8 9 10

- Consultar archivos 0 - 10
- Visitar lugares donde vivían mis antepasados 0 - 10
- Conocer familiares 0 - 10
- Consultar archivos 0 - 10
- Visitar lugares donde vivían mis antepasados 0 - 10
- Conocer familiares 0 - 10

1. Grupos y asociaciones genealógicas.

12. ¿Pertenece o ha pertenecido a algún grupo o asociación genealógica?

- Sí
- No (Marque y pase a la pregunta 17)

13. En caso afirmativo, ¿Le ha servido de ayuda para consultar alguna cuestión genealógica?

- Sí
- No

14. Indique el o los motivos para su consulta a una asociación o grupo genealógico:

- Origen de apellidos y heráldica
- Existencia de antepasados compartidos con otras personas
- Información de archivos (horarios, localización, contactos...)
- Localizaciones geográficas (edificios históricos localidades, museos, comercios…)
- Contacto con familiares lejanos (personas de ascendencia cercana)
- Procedimientos de aprendizaje para investigación genealógica (consejos sobre investigación)

15. Valore la satisfacción sobre la información recibida de la asociación o grupo genealógico (siendo 0 la peor y 10 la mejor valoración):

0 1 2 3 4 5 6 7 8 9 10

- Origen de apellidos y heráldica 0 - 10
- Existencia de antepasados compartidos con otras personas 0 - 10
- Información de archivos 0 - 10
- Localizaciones geográficas 0 - 10
- Contacto con familiares lejanos 0 - 10
- Procedimientos de aprendizaje para investigación genealógica 0 - 10
- Origen de apellidos y heráldica 0 - 10
- Existencia de antepasados compartidos con otras personas 0 - 10
- Información de archivos 0 - 10
- Localizaciones geográficas 0 - 10
- Contacto con familiares lejanos 0 - 10
- Procedimientos de aprendizaje para investigación genealógica 0 - 10

16. Indique el grado de satisfacción en cada uno de los siguientes aspectos relativos a las fuentes de información que le han ayudado a para preparar sus viajes:

0 1 2 3 4 5 6 7 8 9 10

- Familia y amigos 0 - 10
- Agencia de viajes 0 - 10
- Medios de comunicación tradicionales 0 - 10
- Grupos y asociaciones genealógicas 0 - 10
- Redes sociales 0 - 10
- Familia y amigos 0 - 10
- Agencia de viajes 0 - 10
- Medios de comunicación tradicionales 0 - 10
- Grupos y asociaciones genealógicas 0 - 10
- Redes sociales 0 - 10

17. Por favor, indique cuánto dinero se suele gastar en sus viajes (incluyendo transportes, alojamientos, comida ...):
