# Supplementary material for: The sources of information of the genealogical tourist: the influence of social networks and genealogical associations
Source: Heliyon. 2022 Nov 10;8(11):e11551. doi: 10.1016/j.heliyon.2022.e11551 (PMC9668519; doi:10.1016/j.heliyon.2022.e11551)
Supplement: SUPPLEMENTARY TABLE 2.docx [file mmc2.docx]

**Anexo 1**

**1) España:** La Real Academia Matritense de Heráldica y Genealogía, la página web de genealogía Tataranietos.com, Hispagen (Asociación de Genealogía Hispana), la asociación Antzinako, la Asociación Cántabra de Genealogía, Societat Catalana de Genealogia, la Asociación Riojana de Genealogía y Heráldica, Grupo de Genealogía de Andalucía Oriental, Grupo de Genealogía de Andalucía Occidental, Grupo de Genealogía de Murcia, Grupo de Genealogía de Aragón, Grupo de Genealogía de Castilla La Mancha, Linajes Malagueños, Genealogía Soria, la Asociación de amigos del Archivo Histórico Diocesano de Jaén, Asociación Canaria de Genealogía e Historia Familiar, Asociación Cultural de Genealogía e Historia de Aragón, Sociedad Iberoamericana de Genealogía e Historia, el equipo de investigación Historia y Genealogía de Córdoba.

**2) Estados Unidos:** Italian Genealogical Group y la North American Basque Organizationsen.

**3) México:** grupo Genealogía de México.

**4) Colombia**: Academia Colombiana de Genealogía.

**5) Puerto Rico:** Sociedad Puertorriqueña de Genealogía y The Puerto Rican/Hispanic Genealogical Society Inc.
